# Genomic and algorithm-based predictive risk assessment models for benzene exposure

**DOI:** 10.3389/fpubh.2024.1419361

**Published:** 2025-01-21

**Authors:** Minyun Jiang, Na Cai, Juan Hu, Lei Han, Fanwei Xu, Baoli Zhu, Boshen Wang

**Affiliations:** ^1^School of Public Health, Nanjing Medical University, Nanjing, Jiangsu, China; ^2^Jiangsu Province Center for Disease Prevention and Control, Institute of Occupational Disease Prevention, Nanjing, Jiangsu, China; ^3^School of Public Health, Southeast University, Nanjing, Jiangsu, China; ^4^Jiangsu Preventive Medical Association, Nanjing, Jiangsu, China; ^5^Center for Global Health, School of Public Health, Nanjing Medical University, Nanjing, Jiangsu, China

**Keywords:** benzene-induced damage, benzene exposure, machine learning, bioinformatics, risk assessment, occupational health

## Abstract

**Aim:**

In this research, we leveraged bioinformatics and machine learning to pinpoint key risk genes associated with occupational benzene exposure and to construct genomic and algorithm-based predictive risk assessment models.

**Subject and methods:**

We sourced GSE9569 and GSE21862 microarray data from the Gene Expression Omnibus. Utilizing R software, we performed an initial screen for differentially expressed genes (DEGs), which was followed by the enrichment analyses to elucidate the affected functions and pathways. Subsequent steps included the application of three machine learning algorithms for key gene identification, and the validation of these genes within both a cohort exposed to benzene and a benzene-exposed mice model. We then conducted a functional prediction analysis on these genes using four machine learning models, complemented by GSVA enrichment analysis.

**Results:**

Out of the data, 40 DEGs were identified, primarily linked to cytokine signaling, lipopolysaccharide response, and chemokine pathways. NFKB1, PHACTR1, PTGS2, and PTX3 were pinpointed as significant through machine learning. Validation confirmed substantial changes in NFKB1 and PTX3 following exposure, with PTX3 emerging as paramount, suggesting its utility as a diagnostic biomarker for benzene damage.

**Conclusion:**

Risk assessment models, informed by oxidative stress markers, successfully discriminated between benzene-injured patients and controls.

## 1 Introduction

Benzene is a ubiquitous environmental and industrial chemical that is classified as a Group I carcinogen by the International Agency for Research on Cancer (44) because of its ability to cause a variety of blood-related diseases. In the occupational setting, benzene exposure occurs primarily in the petroleum, chemical, shoe and paint industries (1). About 500,000 people in China work with benzene or benzene-containing solvents (2), and China's production and use of pure benzene is increasing every year, with production reaching 11.45 million tons in 2016 and supply expected to reach 19.3 million tons by 2023 (3). In order to prevent blood diseases and protect the health of workers who receive benzene, all countries in the world have formulated occupational exposure limits for benzene, and the occupational exposure limits for benzene now implemented in China are higher than the foreign health standards. However, even with the implementation of national standards, chronic low-level benzene exposure could still cause changes in some of the indicators of blood routine (4). This shows that our current occupational exposure limits for benzene do not protect the health of workers who receive benzene (3). Machine learning is understood by some scholars as “a computer program that is able to learn from experience with certain tasks and performance measures (5).” The machine learning process consists of two main steps, the first step is to generate models by analyzing big data; the second step is to draw inferences from the analysis (6). As a core area of artificial intelligence and data science (7), machine learning could easily adapt to new environments and is also self-tuning (8), That's why the application of machine learning in bioinformatics is becoming increasingly popular, and it's being used by researchers to tap into the underlying mechanisms, potential biomarkers, and therapeutic targets of various diseases (9).

Based on this, this study used a bioinformatics approach combining three different machine learning algorithms to obtain characteristic genes for occupational benzene exposure, and then we used four machine models to further analyze the key genes in order to explore the early biomarkers of long-term low-level occupational benzene exposure. Combining early biomarkers with machine learning algorithms could be effective for risk assessment and early identification of benzene poisoning, which provides a theoretical basis for early intervention in benzene toxicity and a more comprehensive and sensitive model for risk assessment.

## 2 Materials and methods

### 2.1 Materials

We downloaded the benzene-induced damage expression profiling datasets GSE9569 and GSE21862 from the GEO database. The GSE21862 dataset includes 83 benzene-connected workers exposed to different benzene concentrations and 42 unexposed controls. Of these, 29 workers exposed to < 1 ppm benzene at most dosimetry reading over a 14-month period (Very Low), 30 exposed to average < 1 ppm (Low), 11 exposed to 5–10 ppm (High), 13 exposed to >10 ppm (Very High). The GSE9569 dataset includes eight benzene-exposed workers and eight non-exposed controls, and the resulting data were compared on two microarray platforms, but the level of exposure in this dataset is unknown.

### 2.2 Diagnostic criteria and populations

The diagnosis of chronic mild benzene poisoning was judged with reference to “the Diagnostic Standard for Occupational Benzene Poisoning” GBZ 68-2022: Occupational history of close exposure to benzene for 3 months or more, which may be accompanied by dizziness, headache, fatigue, insomnia, memory loss, recurrent infections and other clinical manifestations. Review peripheral blood cell analysis every 2 weeks for 3 months with one of the following criteria: (a) White blood cell count below 3.5 × 10^9^/L on 4 or more occasions (see WS/T 405); (b) Neutrophil count below 1.8 × 10^9^/L on 4 or more occasions (see WS/T 405); (c) Platelet count below 80x10^9^/L on 4 or more occasions.

Based on the above diagnostic criteria, we intend to establish a five-year prospective cohort of benzene-exposed workers. In the first year, we selected 445 study participants, 214 in the benzene-exposed group and 231 in the benzene-non-exposed group, and analyzed the general condition of the study participants in relation to their blood counts.

### 2.3 Methods

#### 2.3.1 Data processing and analysis of variances

We eliminate the extrinsic differences between the two datasets by principal components analysis (PCA); We used the R software “limma” package to screen the differentially expressed genes between GSE9569 and GSE21862 with *P* < 0.05 and |logfoldchange| ≥ 0.5 as the initial screening condition, after that, we use the “pheatmap” package to plot heat maps of DEGs and the “ggplot2” package to plot volcano maps of DEGs.

#### 2.3.2 Enrichment analysis of DEGs

We used the “ClusterProfiler” package in R software for GO, KEGG and GSEA enrichment analysis of DEGs. The GO analysis includes cell components (CC), molecular function (MF) and biological process (BP) (10). A standard of *p* < 0.05 was used and bubble plots were used to show the results of the enrichment analysis.

#### 2.3.3 Screening and validation of key genes

We screened the feature genes through R software using three machine learning algorithms: lasso regression algorithm, SVM-RFE support vector machine recursive feature elimination algorithm and random forest algorithm, and then took the intersection of these by employing venn package to obtain the key genes. The R software was used to plot the receiver operating characteristic curve (ROC) for the key genes obtained, and AUC was calculated separately, with larger AUC values indicating better diagnostic performance; and violin plots were made.

#### 2.3.4 Population validation and statistical analysis

Data were analyzed using SPSS 27.0 software, and for information that conformed to a normal distribution, $\bar{x}\pm s$was used to indicate, comparisons of differences between groups were analyzed using independent samples *t*-tests; for information that did not fit a normal distribution, the median and quartiles were expressed, and non-parametric tests were used to compare differences between groups. *p* < 0.05 indicates that the difference is significant.

#### 2.3.5 Modeling of benzene-exposed mice

We chose clean-grade C57BL/6 male mice provided by the animal laboratory of Nanjing Medical University to construct this benzene-exposed mouse model. After numbering, weighing, and excluding underweight or overweight mice, we randomly numbered the mice according to the office software, and divided them into 2 groups (*n* = 12), with corn oil as solvent, and set up solvent control and benzene 150 mg/(kg-b.w.) groups, respectively. The difference in body weight of mice should not exceed 10% of the average body weight of all mice, and the difference in average body weight between groups should not exceed 5%. We took the subcutaneous injection method to stain the mice, and chose to perform the injection once every morning for 5 consecutive days, with an interval of 2 days in between, and the staining time was 4 weeks in total, which started on the 5th day and ended on the 34th day. Mice were executed 24 h after the last administration of benzene corn oil or corn oil alone, and then blood was collected from the orbital venous plexus as well as urine specimens for the next step of analysis and manipulation.

#### 2.3.6 Mouse blood test

We used orbital venous plexus for blood collection from mice. Subsequently, peripheral blood hematological indices, including neutrophils (ANC), platelets (PLT) and white blood cells (WBC), were measured in C57BL/6 mice.

#### 2.3.7 Detection of related substances in mouse urine

Detection of 8-hydroxy-deoxyguanosine (8-OHdG) and S-phenylmercapturic acid (S-PMA) in mouse urine. In this experiment, we chose 8-hydroxydeoxyguanosine quantification kit from Nanjing Sembega Biotechnology Co, 8-OHdG detection kit was chosen to determine the content of 8-OHdG in mouse urine by ELISA double antibody sandwich method. As previously reported (11), We used liquid chromatography-electrospray tandem mass spectrometry for the determination of urinary S-PMA, and the limit of detection was 0.01 mg/L.

#### 2.3.8 Oxidative stress detection

Urine serum malondialdehyde (MDA) concentrations were determined by the OxiSelect DNA oxidative damage ELISA kit (product no. STA-320, Cell Biolabs, Inc., San Diego, CA, USA).

#### 2.3.9 Cell lines and cell cultures

Mouse bone marrow cells were selected for this experiment for subsequent manipulation. Cells were cultured using Iscove's modified Dulbecco's medium (GIBCO, Grand Island, NY, USA) in a 5% CO_2_ incubator at 37°C, It contained 10% fetal bovine serum (FBS, GIBCO), 100 U/mL penicillin and 100 mg/mL streptomycin.

#### 2.3.10 Total RNA extraction and qPCR

This experiment used TRIzol reagent (Gibco, USA) to isolate high-quality total RNA from the cells, which was then treated with Invitrogen SuperScript III reverse transcriptase to generate cDNA. qPCR assays were performed using SYBR Green qPCR MasterMix and Applied Biosystems StepOne qPCR instrument (Carlsbad, CA, USA). β-actin was used as an internal reference. In the analysis of the results, conforming to normally distributed data, expressed as$\bar{x}\pm s$, and comparisons of differences between groups were analyzed using the independent samples *t*-test, the remaining data that did not fit the normal distribution were expressed as medians and quartiles, and comparisons of differences between groups were analyzed using nonparametric tests. *p* < 0.05 represents a significant difference.

### 2.4 Selection and application of machine algorithms

In this study, different models were used to train the screened genes, and the trained models were applied to the test set to obtain the confusion matrices of the different models, and after calculating the accuracy of each model and plotting the ROC curves and making comparisons, the appropriate model was finally obtained for subsequent analysis. Plotting of comparisons using GraphPad Prism 8.3 software. The models applied in this study are Random Forest, Support Vector Machine, bp Neural Network and Bayesian. The models applied in this study are C5.0 Decision Tree (C5.0DT), Support Vector Machine, BP Neural Network and Naïve Bayes.

### 2.5 Gene set variation analysis of key genes

Gene set variation analysis (GSVA) is a non-parametric, unsupervised analysis method that is primarily used to evaluate gene set enrichment results from sequencing (12). In this study, we used the “GSVA” software package to evaluate genome-associated pathways.

## 3 Results

### 3.1 GSE9569 and GSE21862 dataset DEGs

**Figure 1:**
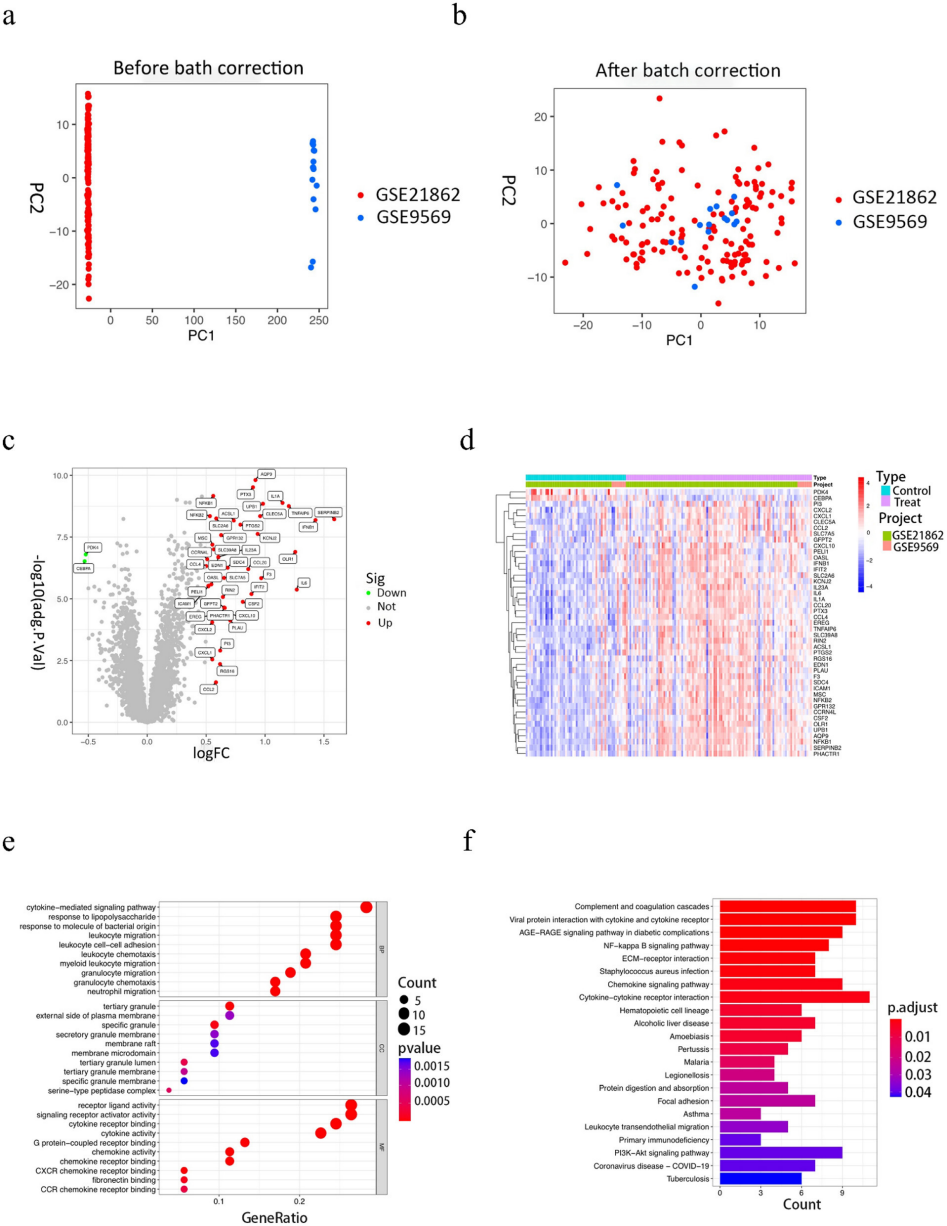
Results of the analysis of the GSE9569 and GSE21862 datasets and functional enrichment results of differential genes. **(A, B)** The 2D plots of the first two components from the principal component analysis results. a is before adjustment and b is after adjustment. As could be seen from the plots, there is a large overlap between the two datasets after adjustment. **(C)** Volcano plot of differentially expressed gene results, with red dots for up-regulated genes, green dots for down-regulated genes, and gray dots for non-differentially expressed genes. **(D)** Heat map of differentially expressed gene clustering. **(E)** Results of GO enrichment analysis, the graph is divided into three sections, from top to bottom, BP biological processes, CC cellular components, and MF molecular functions, with colors corresponding to the degree of enrichment. **(F)** KEGG enrichment analysis results, the horizontal coordinate indicates the number of genes enriched in the pathway, the vertical coordinate indicates the pathway name, and the color corresponds to the degree of enrichment.

After eliminating the extrinsic differences between the two datasets by PCA analysis (Figures 1A, B), differential analysis screened a total of 40 DEGs, of which 38 were up-regulated genes and 2 were down-regulated genes, mapping volcano and heat maps. The volcano plot demonstrates the differential genes of GSE9569 and GSE21862, with up-regulated genes in red and down-regulated genes in green (Figure 1C); the clustered heatmap shows the specific expression of DEGs in each sample (Figure 1D).

### 3.2 Enrichment analysis of DEGs

The screened differential genes were analyzed for GO, KEGG, and GSEA enrichment. GO enrichment analysis revealed (Figure 1E) that bioprocesses (BP) are mainly involved in a series of inflammatory response processes such as cytokine-mediated signaling pathways, leukocyte migration, leukocyte-cell adhesion, and response to lipopolysaccharide; cellular components (CC) are mainly involved in tertiary granules, specific granules, and serine-like peptidase complexes, etc; molecular function (MF) is mainly concerned with cytokine receptor binding, cytokine activity, receptor ligand activity, etc. The results of KEGG enrichment analysis (Figure 1F) showed that the enriched pathways were mainly focused on inflammatory and immune response pathways, such as cytokine-cytokine receptor interactions, interactions of viral proteins with cytokines and cytokine receptors, chemokine signaling pathways, and NF -KB signaling pathways, etc. Further analysis by GSEA enrichment revealed that DEGs were enriched in the high-expression group to a set of five positive genes, mainly involved in cytokine receptor interactions, graft-vs.-host disease, jak stat signaling pathway, leishmaniasis infection, and signaling pathways similar to node receptors (Supplementary Figure 7a); five positive gene sets were enriched in the low-expression group, mainly involved in base excision repair, Huntington's disease, oxidative phosphorylation, Parkinson's disease, and RNA degradation (Supplementary Figure 7b).

### 3.3 Screening and validation of key genes

**Figure 2:**
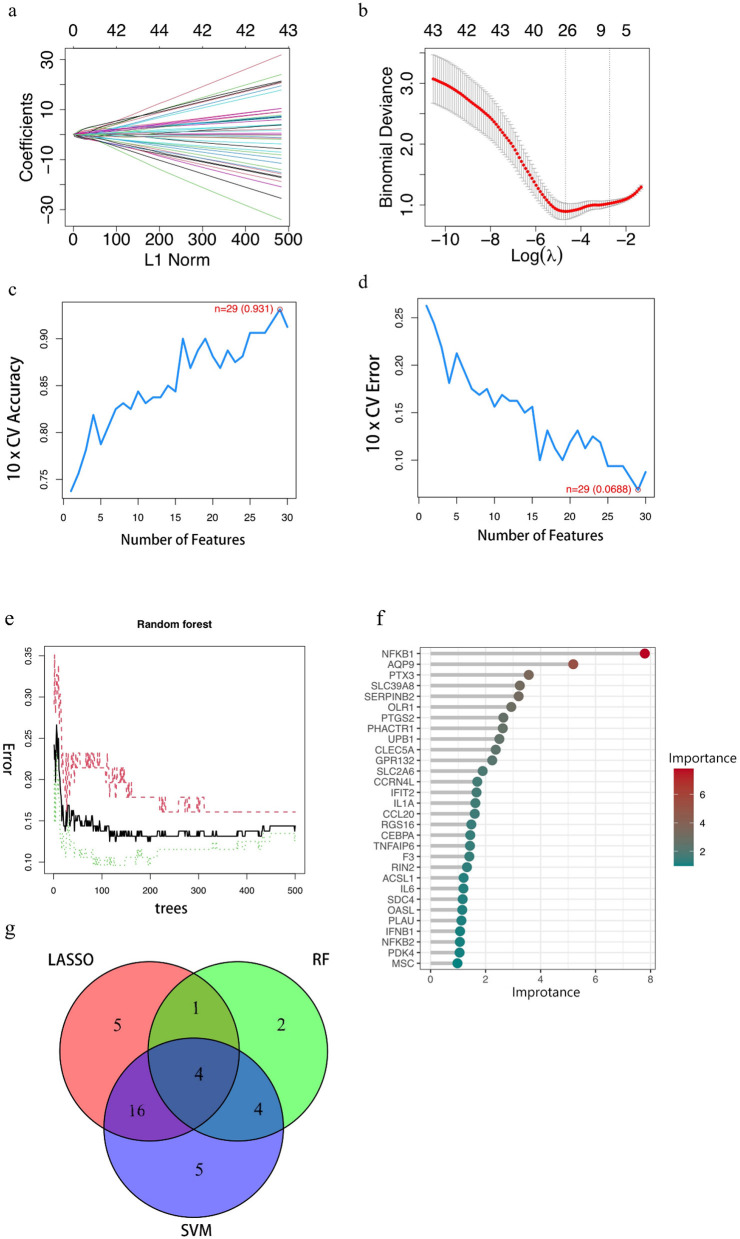
Results of key gene screening**. (A, B)** Identification of benzene-induced damage-related genes using LASSO and cox regression analysis. **(C, D)** The accuracy and the error of the estimate generation for the SVM-RFE algorithm. **(E)** Random forest algorithm. The horizontal coordinate is the number of trees and the vertical coordinate is the cross-validation error; the red line indicates the error in the benzene exposure group, the green line indicates the error in the control group, and the black line indicates the error in all samples. **(F)** Random Forest Gene Importance Ranking. The graph shows the ordering of the top 30 genes in terms of importance. **(G)** Venn diagram demonstrating four genes shared by the LASSO and SVM-RFE and RF algorithms. In the red part, it represents the LASSO algorithm, the green part reaches the standard RF algorithm, the purple part represents the SVM algorithm, the intersection part represents the identified key genes, and the numbers in the figure represent the number of genes.

**Figure 3:**
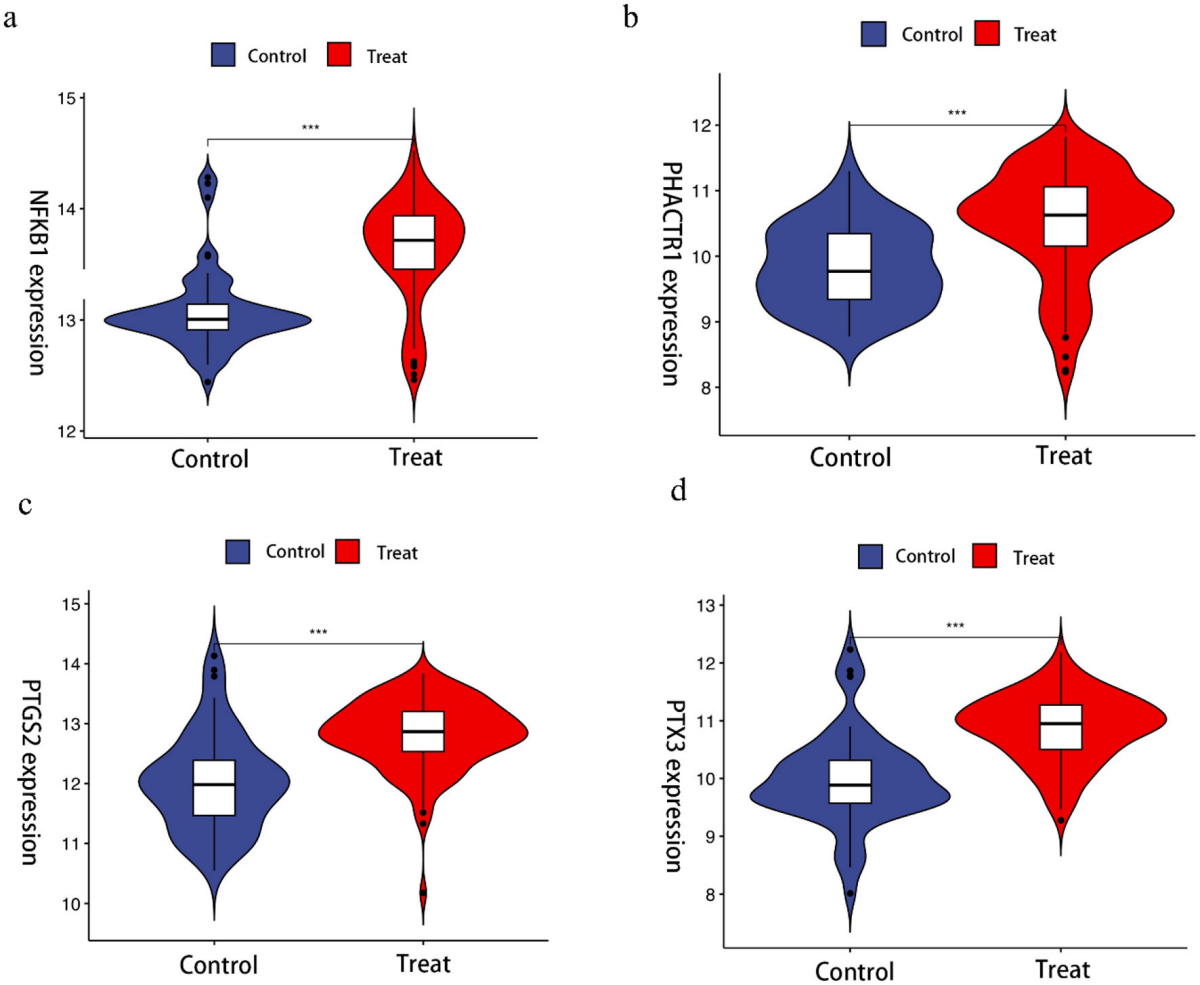
Violin chart showing the relative expression of different genes in Benzene-exposed and non-exposed groups. ^***^Indicates that compared with control group, *p* < 0.001.

The LASSO algorithm was used to screen 26 feature genes from DEGs (Figures 2A, B), the SVM-RFE algorithm was used to screen 29 feature genes (Figures 2C, D), while the RF algorithm was used to screen 11 feature genes (Figures 2E, F), and the intersection of the three was taken to identify four genes (Figure 2G), which are NFKB1, PHACTR1, PTGS2, and PTX3, respectively. For each individual in the abnormal and normal blood group, the level analysis of the four genes was performed (Supplementary Figure 1a); subsequently, ROC curves were plotted for each of the four (Supplementary Figure 2), with AUCs (95% CIs) of 0.822 (0.743–0.893), 0.755 (0.680–0.824), 0.802 (0.714–0.873) and 0.834 (0.756–0.901). We then extracted GEO data to plot violin maps for the four key genes (Figures 3A–D).

### 3.4 Results of population validation and statistical analysis

The results of the basic population analysis for the benzene-exposed and benzene-non-exposed groups are shown in Supplementary Table 1, and the results of the statistical comparison of blood counts with the four genes are shown in Supplementary Figure 3. After comparison, we found that the *p*-values of 8-OHdG, MDA, Neutrophil, PLT, S-PMA, WBC, NFKB1, and PTX3 were all < 0.05, which was statistically significant; while the *p*-values of PHACTR1 and PTGS2 were all >0.05, which was not statistically significant. It could be concluded that the changes in 8-OHdG, MDA, Neutrophil, PLT, S-PMA, WBC, NFKB1 and PTX3 were significant in benzene-exposed group compared to benzene-non-exposed group, and it could not be concluded yet that the changes in PHACTR1 and PTGS2 were significant in benzene-exposed group. Subsequently, according to GBZ 68-2022 “Diagnostic Criteria for Occupational Benzene Poisoning”, the benzene-exposed group was subdivided into the blood abnormality group and the blood normal group (Supplementary Table 2). The results of their statistical comparison were plotted (Supplementary Figure 4). From the results, it could be seen that the *p*-values of PTGS2 and PLT were all >0.05, which was not statistically significant, while the rest of the *p*-values were all < 0.05, which was statistically significant. It could be assumed that the changes in 8-OHdG, MDA, Neutrophil, S-PMA, WBC, NFKB1, PHACTR1 and PTX3 were significant in the abnormal blood group compared to the normal blood group, and it could not be assumed that the changes in PTGS2 and PLT were significant in the abnormal blood group yet. Next, we further analyzed the changes in NFKB1 and PTX3 according to smoking status and sex and found that smoking and sex significantly altered PTX3 (*p* < 0.05), whereas there was no significant effect on NFKB1 (*p* > 0.05) (Supplementary Tables 3, 4). Meanwhile, we analyzed these two genes in GSE21862 and GSE9569, and found that NFKB1 and PTX3 were significantly increased in the benzene-exposed group in GSE21862 compared to the control group (*p* < 0.05), while NFKB1 was significantly increased in the benzene-exposed group in GSE9569 (*p* < 0.05), and the change in PTX3 could not yet be considered significant (*p* > 0.05). Then, this study also analyzed the changes of NFKB1 and PTX3 at different exposure levels in GSE21862, and found that the changes of NFKB1 between the ≪ 1 ppm group and the < 1 ppm group as well as the changes of PTX3 between the < l ppm and 5–10 ppm groups were significant (*p* < 0.05) while the changes of the two genes between the rest of each two groups were not significant (*p* > 0.05) (Supplementary Figure 5).

### 3.5 Four genes differed in benzene-exposed mice vs. control mice

From Supplementary Table 5, it could be seen that the *p*-values of PLT, PHACTR1 and PTGS2 are all >0.05, which is not statistically significant, while the rest of the *p* < 0.05, which is statistically significant. It could be assumed that the changes in S-PMA, 8-OHdG, MDA, WBC, ANC, NFKB1 and PTX3 were significant in benzene-exposed mice compared to those in benzene-non-exposed group, and it could not yet be assumed that the changes in PLT, PHACTR1 and PTGS2 were significant. Plotting the results of their statistical comparison (Supplementary Figure 6).

### 3.6 Determination of machine algorithms

**Figure 4:**
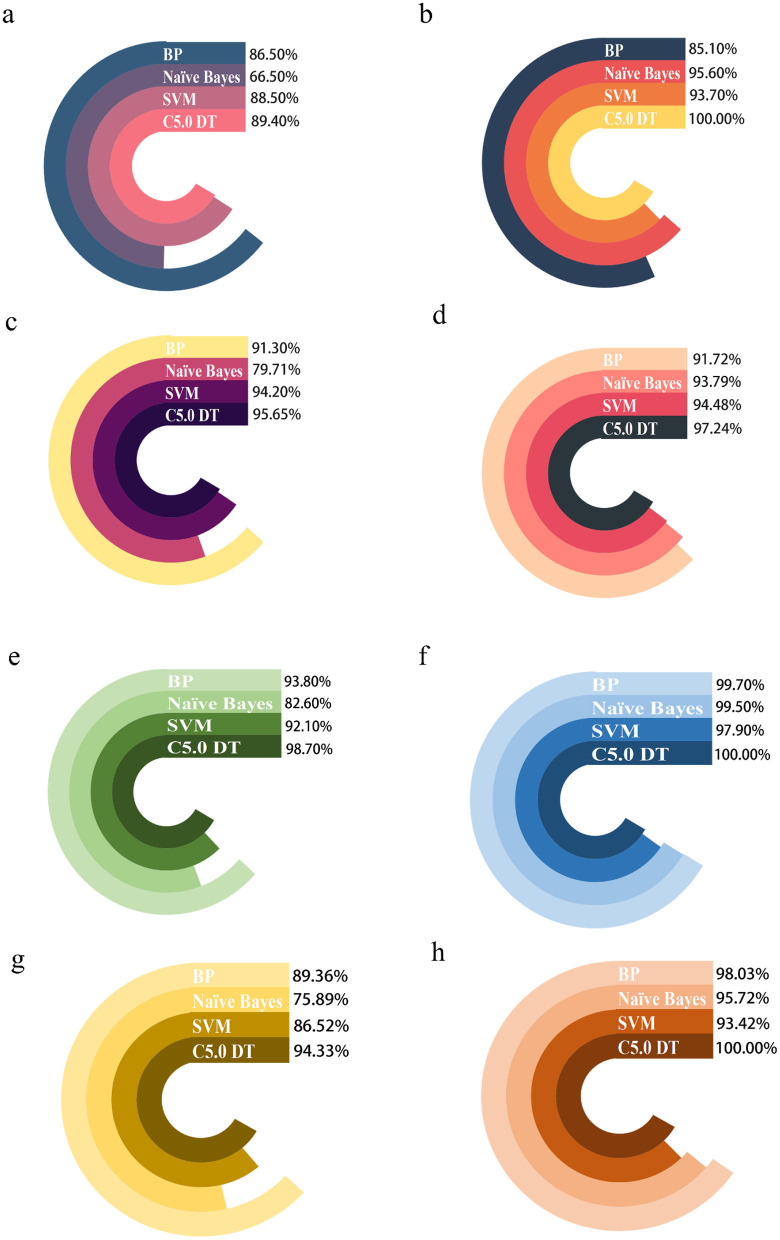
Comparing the predictions of four machine algorithms for benzene-exposed and benzene-non-exposed groups. **(A, C)** are the AUC and accuracy of the test group, and **(B, D)** are the AUC and accuracy of the training group, respectively, **(E, G)** are the AUC and accuracy of the test group and **(F, H)** are the AUC and accuracy of the training group, respectively.

**Figure 5:**
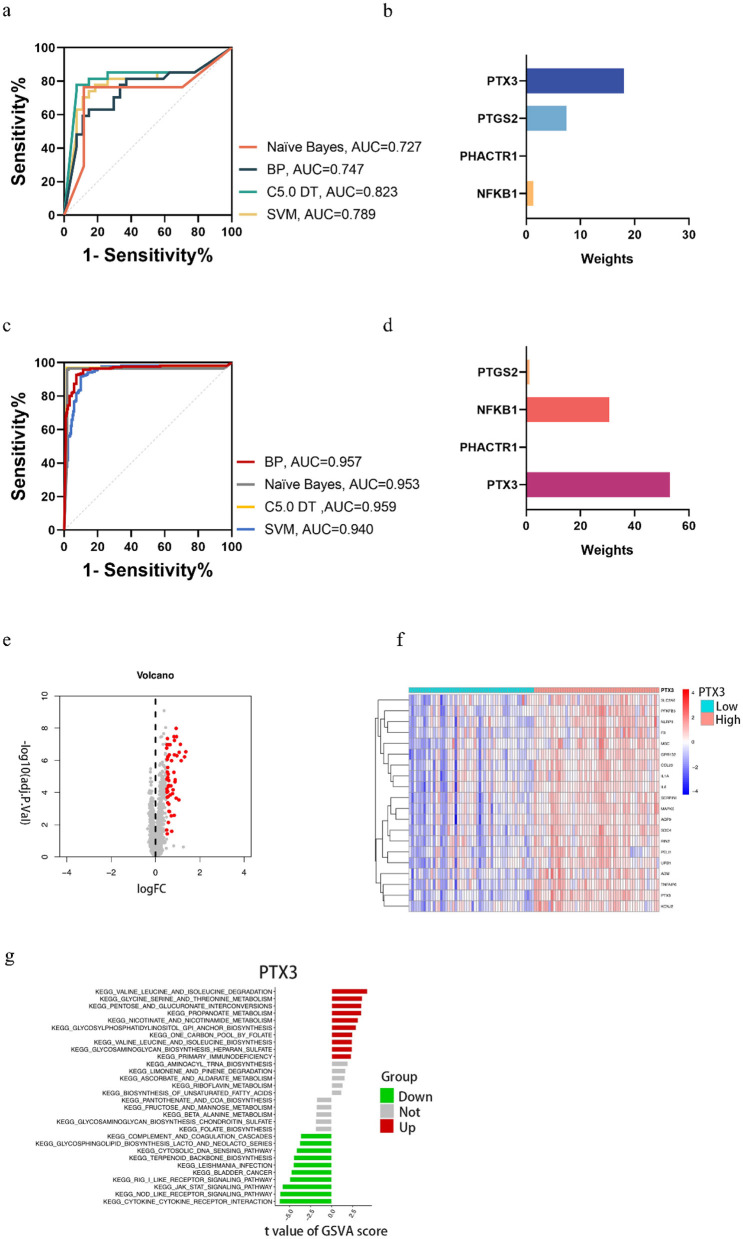
In the benzene-exposed group, **(A)** ROC plots of the four machine models, and **(B)** weight plots of the four genes in the decision tree model; in the benzene-non-exposed group, **(C)** ROC plots of the four machine models, and **(D)** weight plots of the four genes in the decision tree model. And volcanic and heat maps of PTX3 **(E, F)** and results of GSVA analysis of PTX3 **(G)**.

In this study, GraphPad Prism 8.3 software was used to plot the results obtained from different predictive models in the training and test sets. Figure 4 shows that model performance, measured in terms of accuracy and AUC values, is combined with a ROC plot (Figure 5) to compare the four predictive models, C5.0 Decision tree allows better prediction of benzene-induced damage; the prediction results for the benzene-exposed group showed an AUC value and a correct prediction rate of 100% and 97.24%, respectively, for the training group, while the AUC value and prediction accuracy of the training group in the non-benzene-exposed group were both 100%. The weights of the C5.0 DT model were then plotted (Figures 5B, D), according to which PTX3 was the most important gene. In turn, the volcano and heat maps of PTX3 are plotted (Figures 5E, F). Details of the overview of the four machine learning algorithms are given in Supplementary Note 1.

### 3.7 Results of gene set variation analysis

The relevant pathways in different risk populations were further explored for PTX3. Figure 5G shows that in the up-regulated group, PTX3 triggered valine and isoleucine degradation, glycine serine and threonine metabolism, and pentose and glucuronide interconversion, among others; in contrast, PTX3 in the down-regulated group triggered cytokine receptor interactions, node-like receptor signaling pathways, and the jak stat signaling pathway, etc.

## 4 Discussions

**Figure 6:**
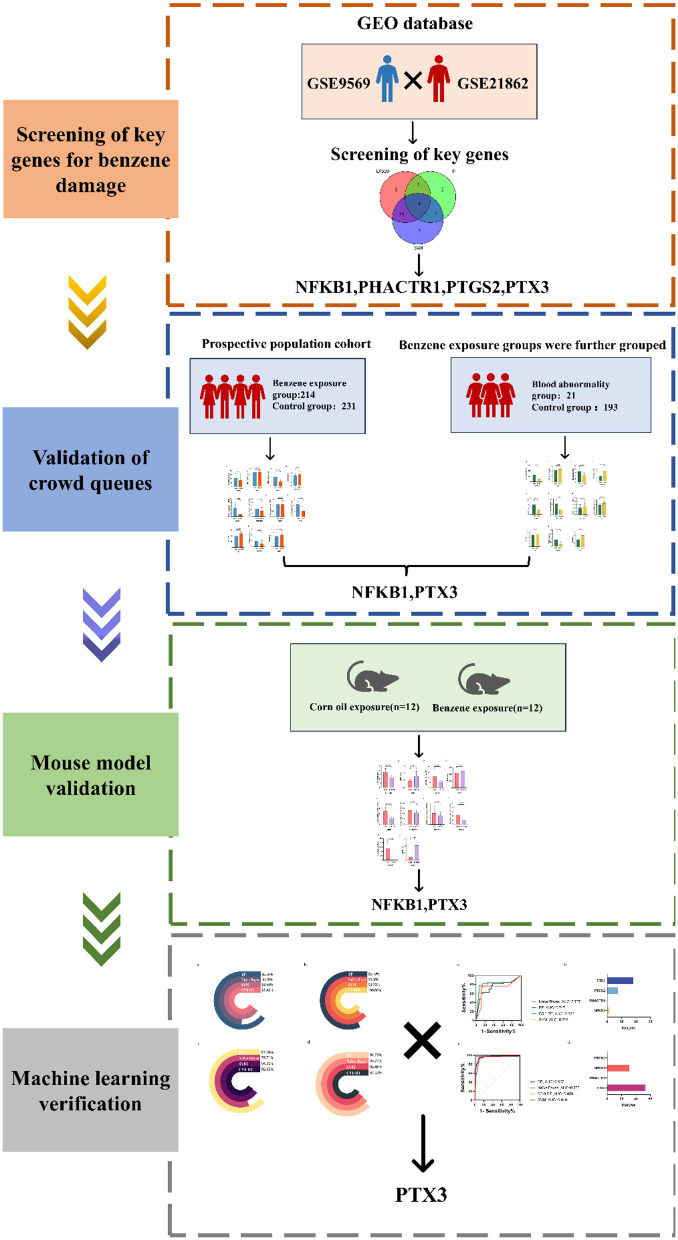
Schematic view of methods.

Benzene is a known and carcinogenic environmental toxicant. As society develops, benzene poisoning is concentrated in occupational settings. Occupational benzene poisoning is categorized into acute and chronic poisoning, and the damage caused to the human body varies. The most common type is occupational chronic benzene poisoning. It is well known that high benzene exposure could cause serious hematologic disorders such as pancytopenia, aplastic anemia and leukemia (13, 14). It has also been shown that chronic low-level benzene exposure induces hematologic toxicity (15–17). Benzene has no safe exposure limit and poses a risk of hematologic malignancies even at relatively low benzene exposure levels (18). Therefore, it is particularly important to actively explore biomarkers for low-level benzene exposure (Figure 6).

Machine learning methods have been very widely and successfully applied in bioinformatics by virtue of their easily adaptable and self-tunable characteristics. genes by three machine learning algorithms: lasso regression algorithm, SVM-RFE support vector machine recursive feature elimination algorithm, and random forest algorithm, and then the intersection of the three screened feature genes was taken, and then four more accurate key genes of benzene-induced damage were obtained: NFKB1, PHACTR1, PTGS2, and PTX3.

We then further validated this with an established population cohort, and in the early phase, we found a strong correlation between two genes, NFKB1 and PTX3, and oxidative stress injury; meanwhile, we found significant differences in the changes of oxidative stress indicators MDA and 8-OhdG as well as intra-benzene exposure indicator S-PMA in benzene-exposed and control groups (*P* < 0.05), which side by side proved the relationship between two genes, NFKB1 and PTX3, and oxidative stress damage. Then we referred to GBZ 68-2022 “Diagnostic Criteria for Occupational Benzene Poisoning” and divided the benzene-exposed group into the blood abnormal group and the blood normal group. Upon analysis, we found that MDA, 8-OhdG and S-PMA were more significantly altered, and the differences in the changes in leukocyte counts and neutrophil counts were also differential (*P* < 0.001). We also found that the differences in the changes in the expression of NFKB1, PHACTR1 and PTX3 were also more pronounced (*P* < 0.05).

To further verify whether these four genes are critical genes for benzene exposure injury, we established a benzene-exposed mouse model and found that the number of neutrophils and the number of leukocytes were significantly lower in benzene-exposed mice compared to mice in the normal group (*P* < 0.05). Benzene exposure is known to disrupt the balance between ROS production and clearance, resulting in oxidative stress (19). MDA, the end product of lipid peroxidation, is commonly used as a measure of oxidative stress and inflammation in biological materials (20). In the present study, we found that benzene-exposed workers and benzene-exposed mice had higher MDA levels than controls; therefore, oxidative stress induced by benzene exposure may play a role in benzene-induced hematotoxicity, which is consistent with previous reports (11). 8-OHdG is one of the oxidative metabolites and is considered a marker of oxidative DNA damage (21). In this study, 8-OhdG was higher in benzene-exposed workers and benzene-exposed mice than in controls, suggesting that benzene exacerbates oxidative damage and causes DNA damage. Compared to other markers, urinary S-PMA is considered to be a biomarker that better demonstrates benzene exposure below 1 ppm (22). In this study, the S-PMA of benzene-exposed workers and benzene-exposed mice was significantly higher than that of the control group, so the change of S-PMA in urine could indirectly reflect the change of benzene concentration in the environment.

Also, the benzene-exposed mouse model showed differential changes in NFKB1 and PTX3 (*P* < 0.05). Combining the three validation results, it could be concluded that NFKB1 and PTX3 are the key genes for benzene exposure injury. As a member of the NF-KB family of transcription factors, NFKB1 plays a central role in benzene hematotoxicity (23, 24). An animal study showed altered myelopoiesis in Nfkb-/- mice, resulting in a significant decrease in bone marrow progenitor cells (25), this side by side validates the central role of NFKB1 in benzene hematotoxicity. The NF-kB pathway leads to post-inflammatory tissue fibrosis (26). This is consistent with the KEGG enrichment analysis we derived above. PTX3, a member of the long pentraxin family, is rapidly produced by phagocytes and stromal cells at sites of inflammation in response to infection or tissue injury (27). PTX3 is induced by oxidative stress (28), sustained oxidative stress leads to chronic inflammation (29). Primary pro-inflammatory signals (bacterial products, interleukin-1 and TNF) produced by different cell types (mainly macrophages and vascular endothelial cells) lead to the production of PTX3 by vascular endothelial cells and macrophages, which is a true and direct indicator of inflammation (30–32). Previous studies have shown that cigarette smoke induces PTX3 expression (33–35); also, PTX3 levels are associated with female fertility (36, 37), which is consistent with our analysis of PTX3 in smoking status and sex. Chronic inflammation and immunosuppression are two key features of benzene (38). Regarding the relationship between chronic inflammation and immunosuppression, numerous scholars have suggested that chronic inflammation induces local immunosuppression (38–43). In general, benzene appears to activate the innate immune system to cause inflammation, but suppresses the adaptive immune system, which is critical for long-term defense and protection against repeated exposure to irritants such as benzene (38).

We note that this study has a number of shortcomings. First, we did not specifically explore the exact link between the two genes, NFKB1 and PTX3, and benzene exposure, and there are some inflammatory conditions that can cause changes in these two genes (e.g., autoimmune diseases), and second, the level of benzene exposure of GSE9569 used for the analysis is unknown, and does not allow us to validate the changes of the two genes in GSE21862 across different exposure levels. Meanwhile, due to the insufficiency of experimental equipment, we were unable to perform strict inhalation benzene exposure, but used subcutaneous injection instead, which may produce some discrepancies in the experimental results. To address these shortcomings, we will conduct more cellular and mouse experiments to supplement the conclusions of this study.

## 5 Conclusions

Conclusively, this investigation amalgamated bioinformatics with machine learning to elucidate pivotal risk genes implicated in benzene-induced damage, unearthing two genes, NFKB1 and PTX3, potentially contributing to benzene's hematotoxicity through inflammatory response. At present, the bulk of research is centered around the interplay between NFKB1 and benzene exposure, with a dearth of studies exploring the correlation between PTX3 and benzene exposure. Our study confirmed alterations in PTX3 following benzene exposure, paving the way for ensuing research, which may focus on the patterns and mechanisms of PTX3 changes post benzene exposure.

The integration of validated key risk genes with machine learning algorithms enables precise assessment of benzotoxicity risk and early identification of symptomatic patients. Our detection of key risk genes offers insights into the mechanisms of benzotoxicity, reflecting the connection between oxidative stress and inflammation. Concurrently, the risk assessment model, constructed on the foundation of key risk genes and machine learning algorithms, serves as an invaluable tool for early screening of high-risk patients, facilitating timely, in-depth examinations and interventions.

## Data Availability

The original contributions presented in the study are included in the article/Supplementary material, further inquiries can be directed to the corresponding authors.

## References

[1] Jie L Huiyao L Wen C. Advances in exposure and effect biomarkers of low-dose benzene. J Med Mol Biol. (2016) 13:360–4. 10.3870/j.issn.1672-8009.2016.06.000

[2] Pei L Xin W Qiang Z. Research progress on biomarkers of occupational benzene exposure. Occupation and Health. (2022) 38:3133–7. 10.13329/j.cnki.zyyjk.2022.0620

[3] Zhang H Li H Peng Z Cao J Bao J Li L. Meta-analysis of the effect of low-level occupational benzene exposure on human peripheral blood leukocyte counts in China. J Environ Sci. (2022) 114:204–10. 10.1016/j.jes.2021.08.035 35459485

[4] Li-Jing H Bi-Kun YU Zhi-Wei G Mei-An HE. Study on the Health Hazard of Low Level Occupational Benzene Exposure. Practical Preventive Medicine (2015). pp. 978–980. 10.3969/j.issn.1006-3110.2015.08.027

[5] Mitchell TM. Machine Learning. (1997).

[6] Jordan MI Mitchell TM. Machine learning: trends, perspectives, and prospects. Science. (2015) 349:255–60. 10.1126/science.aaa8415 26185243

[7] Pappada SM. Machine learning in medicine: it has arrived, let's embrace it. J Card Surg. (2021) 36:4121–4. 10.1111/jocs.15918 34392567

[8] Tan AC Gilbert D. Machine Learning and Its Application to Bioinformatics: An Overview. Glasgow: Department of Computing, University of Glasgow. (2003).

[9] Shastry KA Sanjay H. Machine learning principles for bioinformatics techniques and applications. In: Machine Learning for Bioinformatics. (2020), 25–39. 10.1007/978-981-15-2445-5_3

[10] Gene Ontology Consortium. The gene ontology (GO) project in 2006. Nucleic Acids Res. (2006) 34:D322–D326. 10.1093/nar/gkj021 16381878 PMC1347384

[11] Wang B Xu S Sun Q Li X Wang T Xu K. Let-7e-5p, a promising novel biomarker for benzene toxicity, is involved in benzene-induced hematopoietic toxicity through targeting caspase-3 and p21. Ecotoxicol Environ Saf. (2022) 246:114142. 10.1016/j.ecoenv.2022.114142 36193590

[12] Zhang D Wang M Peng L Yang X Li K Yin H. Identification and validation of three PDAC subtypes and individualized GSVA immune pathway-related prognostic risk score formula in pancreatic ductal adenocarcinoma patients. J Oncol. (2021) 2021:4986227. 10.1155/2021/4986227 34987579 PMC8723862

[13] Koh D-H Jeon H-K Lee S-G Ryu H-W. The relationship between low-level benzene exposure and blood cell counts in Korean workers. Occup Environ Med. (2015) 72:421–7. 10.1136/oemed-2014-102227 25575529

[14] Wang X Zhou J Han L Cheng X Shao H Jia Q. The distribution and concentration monitoring of benzene industries—six PLADs, China, 2020. China CDC Wkly. (2021) 3:897. 10.46234/ccdcw2021.220 34745687 PMC8563330

[15] Casale T Sacco C Ricci S Loreti B Pacchiarotti A Cupelli V. Workers exposed to low levels of benzene present in urban air: assessment of peripheral blood count variations. Chemosphere. (2016) 152:392–8. 10.1016/j.chemosphere.2016.01.096 27011318

[16] Li J Xing X Zhang X Liang B He Z Gao C. Enhanced H3K4me3 modifications are involved in the transactivation of DNA damage responsive genes in workers exposed to low-level benzene. Environm Pollut. (2018) 234:127–35. 10.1016/j.envpol.2017.11.042 29175474

[17] Stenehjem JS Kjærheim K Bråtveit M Samuelsen SO Barone-Adesi F Rothman N. Benzene exposure and risk of lymphohaematopoietic cancers in 25 000 offshore oil industry workers. (2015) 112;1603–12. 10.1038/bjc.2015.108 25867262 PMC4453669

[18] Swaen GM van Amelsvoort L Twisk JJ Verstraeten E Slootweg R Collins JJ. Low level occupational benzene exposure and hematological parameters. Chem Biol Interact. (2010) 184:94–100. 10.1016/j.cbi.2010.01.007 20074561

[19] Liang B Zhong Y Chen K Zeng L Li G Zheng J. Serum plasminogen as a potential biomarker for the effects of low-dose benzene exposure. Toxicology. (2018) 410:59–64. 10.1016/j.tox.2018.09.004 30213540

[20] Busch CJ Binder C. Malondialdehyde epitopes as mediators of sterile inflammation. Biochim Biophys Acta Mol Cell Biol Lipids. (2017) 1862:398–406. 10.1016/j.bbalip.2016.06.016 27355566

[21] Graille M Wild P Sauvain J-J Hemmendinger M Guseva Canu I Hopf NB. Urinary 8-OHdG as a biomarker for oxidative stress: a systematic literature review and meta-analysis. Int J Mol Sci. (2020) 21:3743. 10.3390/ijms21113743 32466448 PMC7313038

[22] Farmer PB Kaur B Roach J Levy L Consonni D Bertazzi PA. The use of S-phenylmercapturic acid as a biomarker in molecular epidemiology studies of benzene. Chem Biol Interact. (2005) 153:97–102. 10.1016/j.cbi.2005.03.013 15935804

[23] Bai W Yang J Yang G Niu P Tian L Gao A. Long non-coding RNA NR_045623 and NR_028291 involved in benzene hematotoxicity in occupationally benzene-exposed workers. Exp Mol Pathol. (2014) 96:354–60. 10.1016/j.yexmp.2014.02.016 24613687

[24] Gao A Yang J Yang G Niu P Tian L. Differential gene expression profiling analysis in workers occupationally exposed to benzene. Sci Total Environ. (2014) 472:872–9. 10.1016/j.scitotenv.2013.11.089 24342094

[25] Cartwright T Perkins ND Wilson C. NFKB1: a suppressor of inflammation, ageing and cancer. FEBS J. (2016) 283:1812–22. 10.1111/febs.13627 26663363

[26] Kiriakidis S Andreakos E Monaco C Foxwell B Feldmann M Paleolog E. VEGF expression in human macrophages is NF-κB-dependent: studies using adenoviruses expressing the endogenous NF-κB inhibitor IκBα and a kinase-defective form of the IκB kinase 2. J Cell Sci. (2003) 116:665–74. 10.1242/jcs.00286 12538767

[27] Doni A Mantovani A Bottazzi B Russo RC. PTX3 regulation of inflammation, hemostatic response, tissue repair, and resolution of fibrosis favors a role in limiting idiopathic pulmonary fibrosis. Front Immunol. (2021) 12:676702. 10.3389/fimmu.2021.676702 34276664 PMC8284251

[28] Wang L Cano M Datta S Wei H Ebrahimi KB Gorashi Y. Pentraxin 3 recruits complement factor H to protect against oxidative stress-induced complement and inflammasome overactivation. J Pathol. (2016) 240:495–506. 10.1002/path.4811 27659908

[29] Reuter S Gupta SC Chaturvedi MM Aggarwal BB. Oxidative stress, inflammation, and cancer: how are they linked? Free Radic Biol Med. (2010) 49:1603–16. 10.1016/j.freeradbiomed.2010.09.006 20840865 PMC2990475

[30] Basile A Sica A d'Aniello E Breviario F Garrido G Castellano M. Characterization of the promoter for the human long pentraxin PTX3: role of NF-κB in tumor necrosis factor-α and interleukin-1β regulation. J Biol Chem. (1997) 272:8172–8. 10.1074/jbc.272.13.8172 9079634

[31] Bottazzi B Vouret-Craviari V Bastone A De Gioia L Matteucci C Peri G. Multimer formation and ligand recognition by the long pentraxin PTX3: similarities and differences with the short pentraxins C-reactive protein and serum amyloid P component. J Biol Chem. (1997) 272:32817–23. 10.1074/jbc.272.52.32817 9407058

[32] Erbas O Pala HG Pala EE Oltulu F Aktug H Yavasoglu A. Ovarian failure in diabetic rat model: nuclear factor-kappaB, oxidative stress, and pentraxin-3. Taiwan J Obstet Gynecol. (2014) 53:498–503. 10.1016/j.tjog.2013.11.008 25510691

[33] Pauwels NS Bracke KR Maes T Pottelberge GRV Garlanda C Mantovani A. Cigarette smoke induces PTX3 expression in pulmonary veins of mice in an IL-1 dependent manner. Respir Res. (2010) 11:134. 10.1186/1465-9921-11-134 20920344 PMC2959025

[34] Poznanski M Brzezianska-Lasota E Kiszalkiewicz J Kurnatowska I Kroczynska-Bednarek J Pekala-Wojciechowska A. Serum levels and gene expression of pentraxin 3 are elevated in COPD. Adv Med Sci. (2019) 64:85–9. 10.1016/j.advms.2018.08.006 30572222

[35] Van Pottelberge GR Bracke KR Pauwels NS Vermassen FE Joos GF Brusselle GG. COPD is associated with reduced pulmonary interstitial expression of pentraxin-3. Eur Respir J. (2012) 39:830–8. 10.1183/09031936.00138110 21920889

[36] Bottazzi B Bastone A Doni A Garlanda C Valentino S Deban L. The long pentraxin PTX3 as a link among innate immunity, inflammation, and female fertility. J Leukoc Biol. (2006) 79:909–12. 10.1189/jlb.1005557 16478917

[37] May L Kuningas M van Bodegom D Meij HJ Frolich M Slagboom PE. Genetic variation in pentraxin (PTX) 3 gene associates with PTX3 production and fertility in women. Biol Reprod. (2010) 82:299–304. 10.1095/biolreprod.109.079111 19846603

[38] Guo H Ahn S Zhang L. Benzene-associated immunosuppression and chronic inflammation in humans: a systematic review. Occup Environ Med. (2021) 78:377–84. 10.1136/oemed-2020-106517 32938756 PMC7960562

[39] Baniyash M. Chronic inflammation, immunosuppression and cancer: new insights and outlook. Semin Cancer Biol. (2006) 16:80–8. 10.1016/j.semcancer.2005.12.002 16420981

[40] Kanterman J Sade-Feldman M Baniyash M. New insights into chronic inflammation-induced immunosuppression. Semin Cancer Biol. (2012) 22:307–18. 10.1016/j.semcancer.2012.02.008 22387003

[41] Li L Yu R Cai T Chen Z Lan M Zou T. Effects of immune cells and cytokines on inflammation and immunosuppression in the tumor microenvironment. Int Immunopharmacol. (2020) 88:106939. 10.1016/j.intimp.2020.106939 33182039

[42] Sun Y Liu N Guan X Wu H Sun Z Zeng H. Immunosuppression induced by chronic inflammation and the progression to oral squamous cell carcinoma. Mediators Inflamm. (2016) 2016:5715719. 10.1155/2016/5715719 28053372 PMC5178366

[43] Wang D DuBois RNJC. Immunosuppression associated with chronic inflammation in the tumor microenvironment. Carcinogenesis. (2015) 36:1085–93. 10.1093/carcin/bgv123 26354776 PMC5006153

[44] WHO. IARC Monographs on the evaluation of the carcinogenic risk of chemicals to humans. Vol. 29: Some Industrial Chemicals and Dyestuffs. (1982). pp. 93–148. 6957379

