# Supplementary material for: Genomic and algorithm-based predictive risk assessment models for benzene exposure
Source: Front Public Health. 2025 Jan 21;12:1419361. doi: 10.3389/fpubh.2024.1419361 (PMC11795664; doi:10.3389/fpubh.2024.1419361)
Supplement: Supplementary file 1 [file Supplementary_file_1.docx]

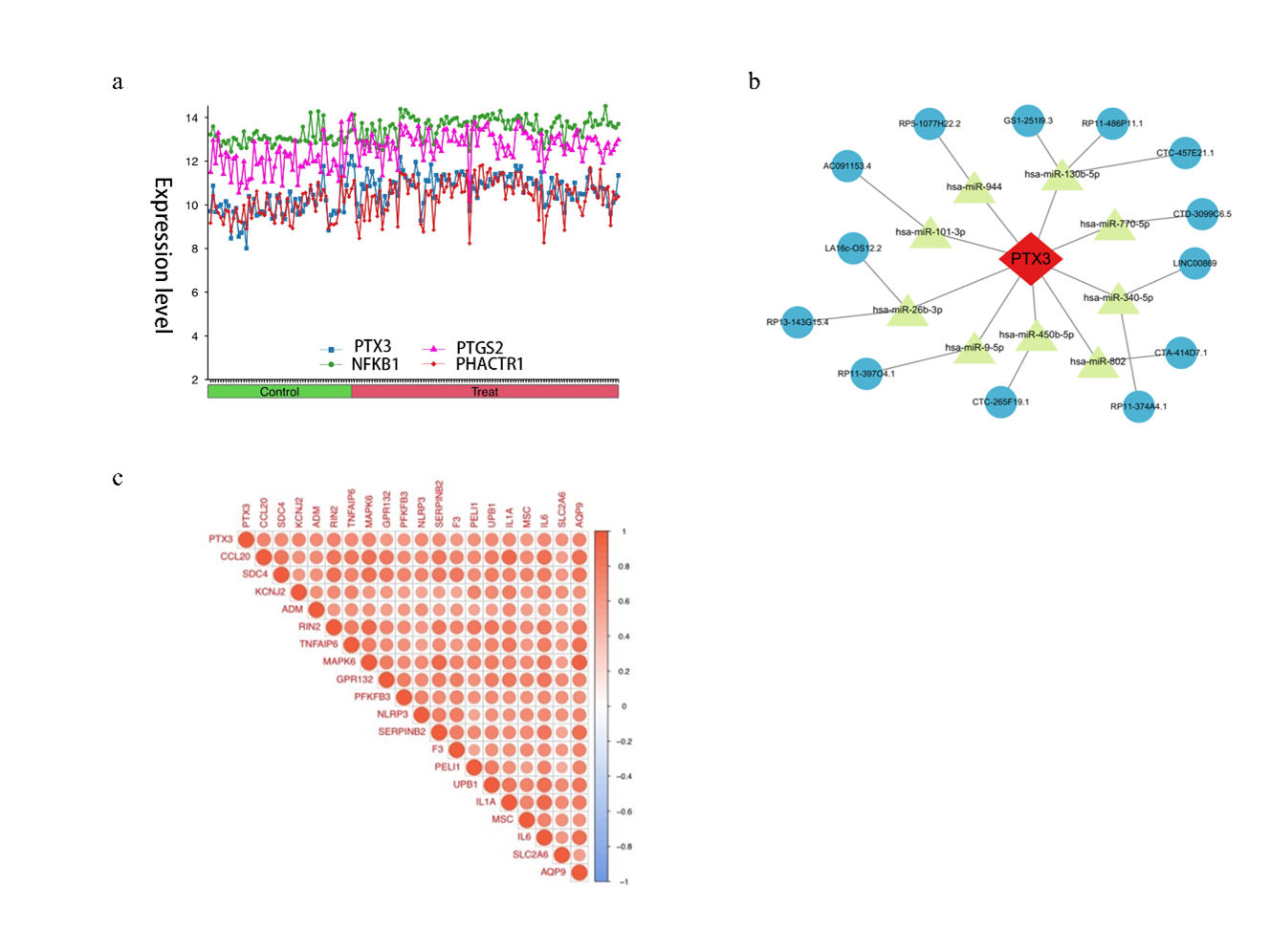


**Fig.1**. Results of analyses of genes. (a) Expression levels of the four genes screened on individuals in the abnormal and normal blood groups. (b) The ceRNA network of lncRNA-miRNA-mRNA of PTX3. The triangles denote miRNAs, circles denote lncRNA, and rhombuses denote mRNA. A total of 1 DELs, 9 DEMs,13 DEGs were included in the network. (c) Distribution of genes related to PTX3.


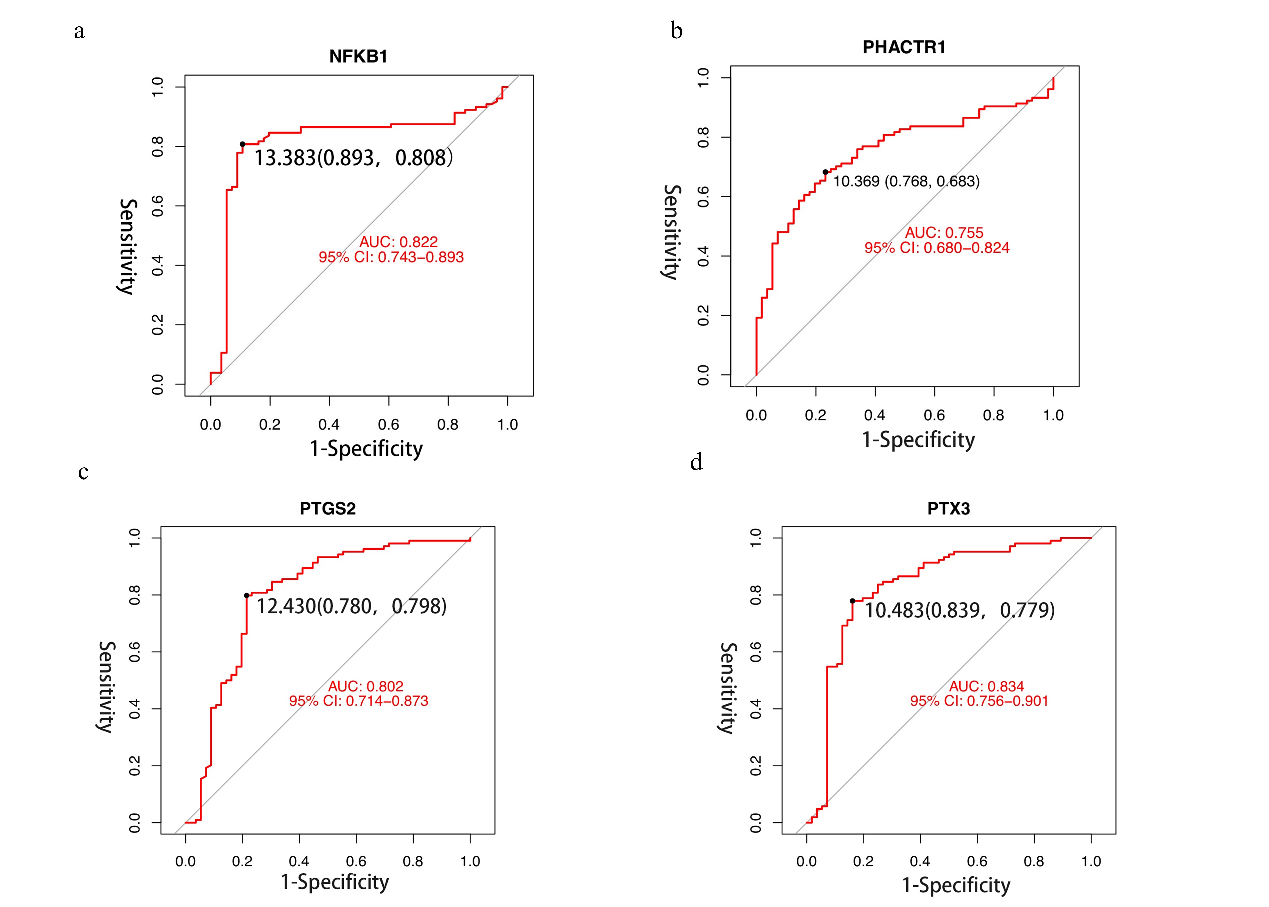


**Fig.2.** AUC plots of the four genes showed that PTX3 was the highest.


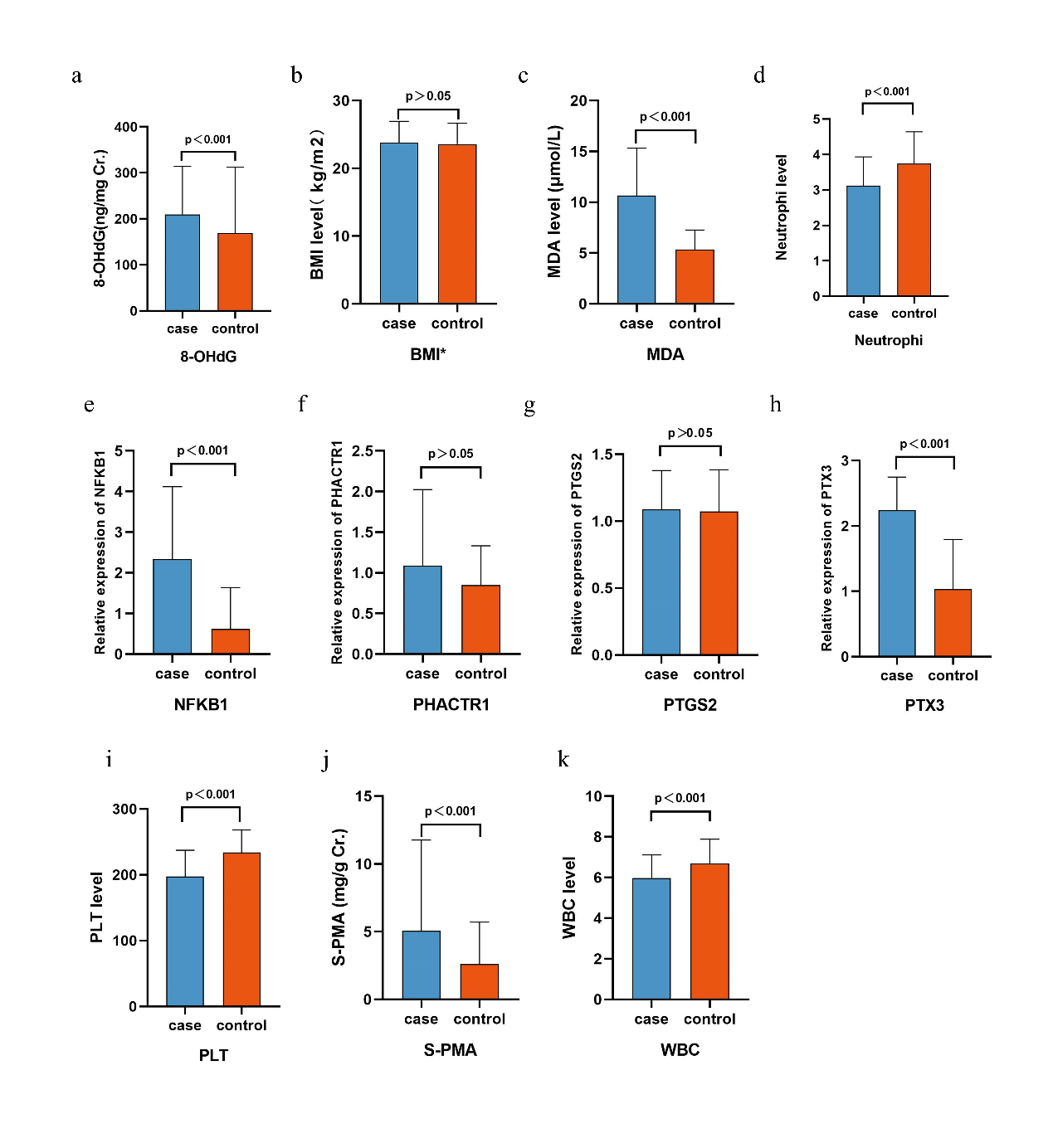


“*”Indicates the use of independent samples t-test.

**Fig.3.** Results of statistical analysis of benzene-exposed and benzene-non-exposed groups.


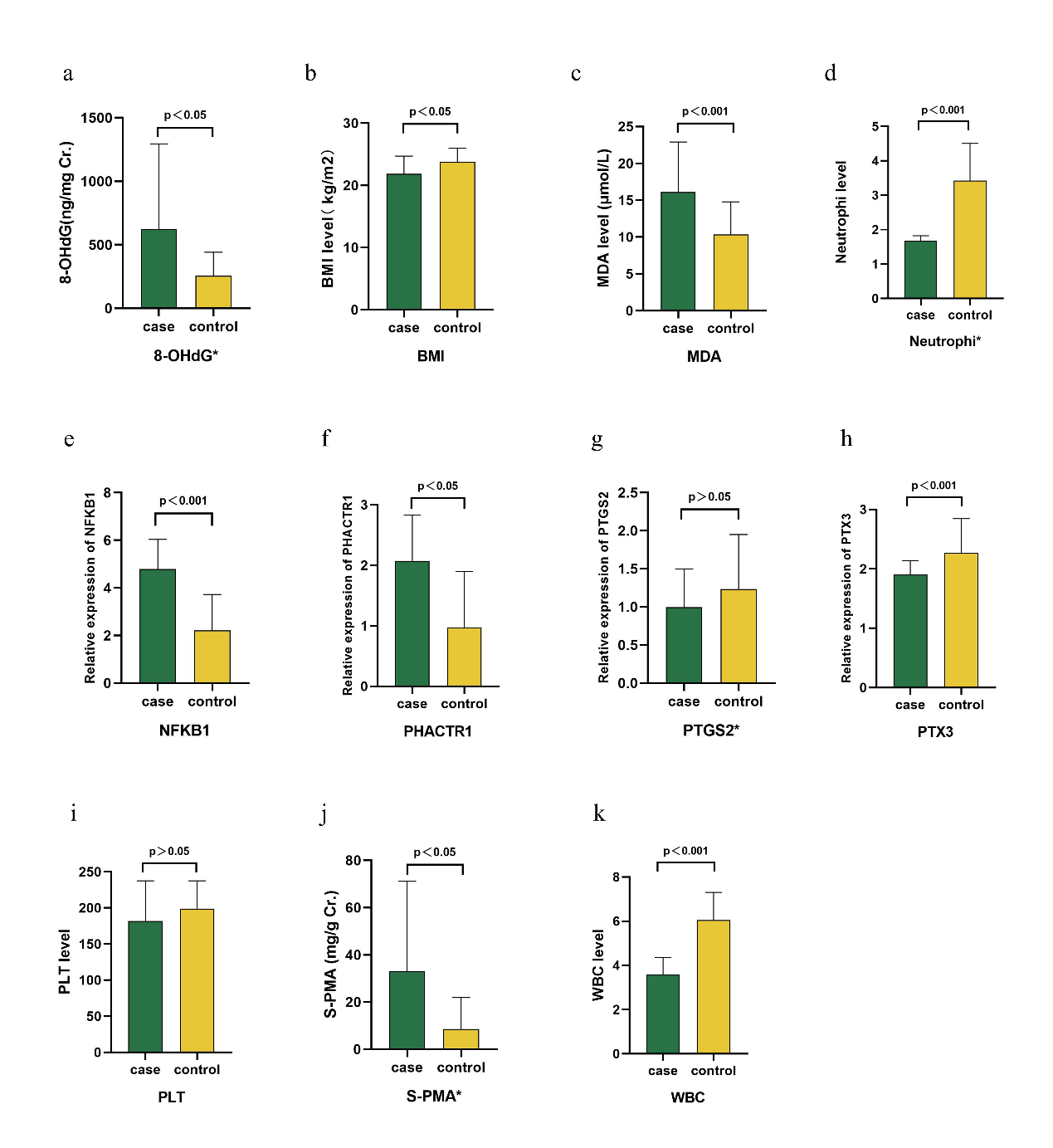


“*”Indicates the use of independent samples t-test.

**Fig.4.** Statistical analysis results of the abnormal blood group and normal blood group.


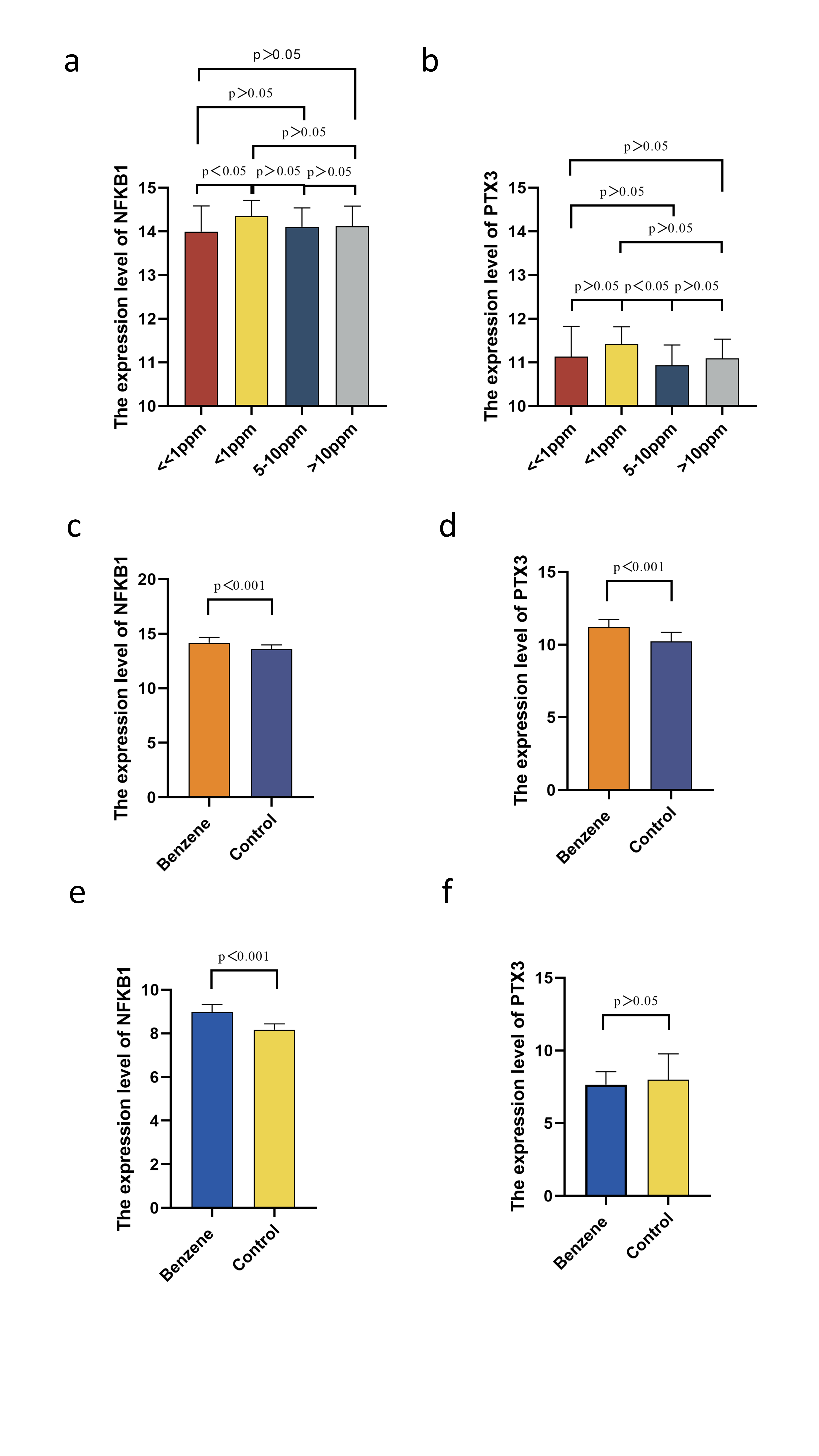


**Fig.5.** Results of NFKB1 and PTX3 analysis in two datasets, GSE21862 and GSE9569. (a) Levels of change in NFKB1 in GSE21862 for different exposure ranges. (b) Levels of change in PTX3 in GSE21862 across different exposure ranges. (c) Changes in NFKB1 between benzene-exposed and control groups in GSE21862. (d) Change in PTX3 between benzene-exposed and control groups in GSE21862. (e) Changes in NFKB1 between benzene-exposed and control groups in GSE9569. (f) Changes in PTX3 between benzene-exposed and control groups in GSE9569.


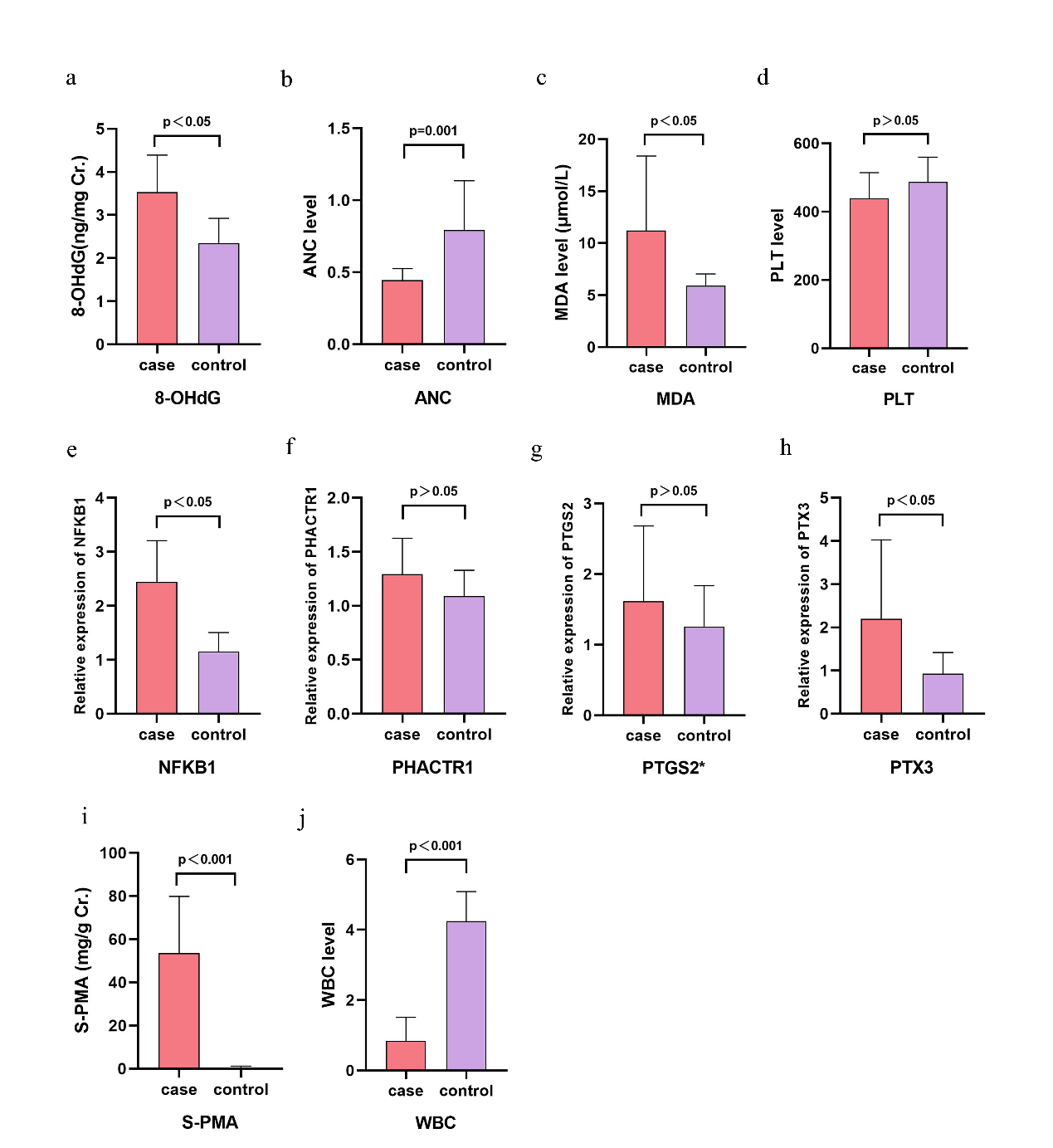


“*”Indicates the use of independent samples t-test.

**Fig.6.** Results of statistical analysis of benzene-exposed mice and controls.


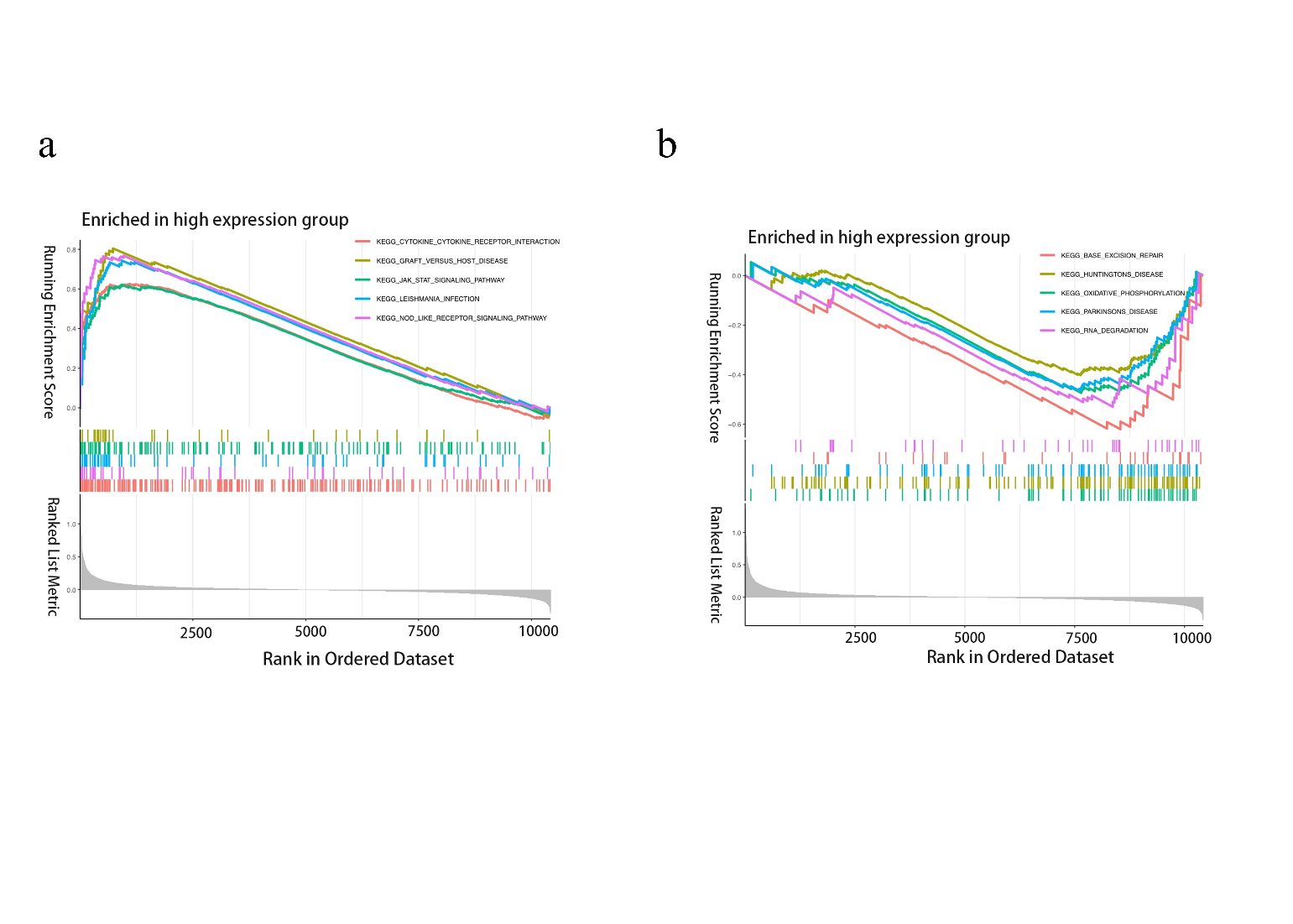


**Fig.7.** Results of GSEA analysis of DEGs. (a) GSEA of DEGs in the high expression group; (b) GSEA of DEGs in the low expression group.
