# Supplementary material for: Genomic and algorithm-based predictive risk assessment models for benzene exposure
Source: Front Public Health. 2025 Jan 21;12:1419361. doi: 10.3389/fpubh.2024.1419361 (PMC11795664; doi:10.3389/fpubh.2024.1419361)
Supplement: Supplementary file 2 [file Supplementary_file_2.docx]

**Genomic and Algorithm-Based Predictive Risk Assessment Models for Benzene Exposure**

Minyun Jiang^a ,1^, Boshen Wang^b,1^, Na Cai^a^, Juan Hu^e^, Lei Han^b,c,^ , Fanwei Xu^e^, Baoli Zhu^a,b,c,d, ,#^

**journal name：Environmental Science and Pollution Research**

1. School of Public Health, Nanjing Medical University, Nanjing 210000, Jiangsu China
2. Institute of Occupational Disease Prevention, Jiangsu Province Center for Disease Prevention and Control, Nanjing 21000, Jiangsu, China.
3. Jiangsu Preventive Medical Association, Nanjing 210000, Jiangsu, China;
4. Center for Global Health, School of Public Health, Nanjing Medical University, Nanjing 210000, Jiangsu, China
5. Southeast University, Nanjing 210009, Jiangsu, China;

# Correspondence to: Baoli Zhu, School of Public Health, Nanjing Medical University, Nanjing 210000, Jiangsu China. Tel: 025-83759982, Fax: 025-83759310, E-mail: [zhublcdc@sina.com;](mailto:zhubl@jscdc.cn;)

1 Minyun Jiang and Boshen Wang have contributed equally to this research. Biographical note of the first author: Minyun Jiang, female, born in 1998, MPH student, majoring in public health; Boshen Wang, male, born in 1991, PD, majoring in public health.

**Overview of Machine Learning Models**

1. Decision tree classification is a machine learning technique that achieves homogeneous classification of target variables by recursively partitioning a data set. In the field of machine learning, decision trees are one of the more basic algorithms that can be used for classification and regression. Decision trees are popular because of their ability to construct graphical models; the principle is to split the dataset from the root node by selecting features, and keep going down until it reaches the leaf nodes, whose decision results are the classification results. As the decision tree division continues, it is often desirable that the branch nodes of the decision tree contain samples that belong to the same class as much as possible. Information gain can be obtained through this process(Wang et al., 2023).


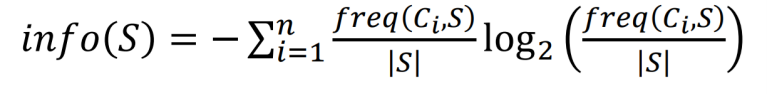
 (1)

and
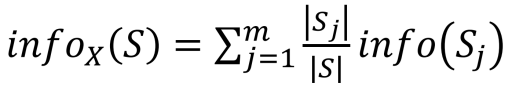
 (2)

Thus, the information gain in equation (3) can be obtained from the equations (1) and (2).


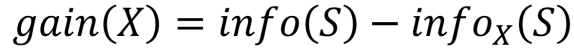
 (3)

1. BP neural network is a multilayer network with one input layer, one or more hidden layers and one output layer. Each neuron on the hidden and output layers corresponds to an excitation function and a threshold value. Changing the weight coefficients of the hidden layers can change the performance of the entire multilayer neural network. The essence of the algorithm is the problem of minimizing the error function.The BP neural network algorithm is divided into two processes: forward propagation and back propagation(Cai-Hong, 2012). In forward propagation the input data is processed layer by layer from the input layer through the hidden layer and then passed to the output layer. If the actual output value of the output layer does not match the desired output value, then it enters into back propagation. Reverse propagation is to reverse the error signal from the hidden layer to the input layer, while modifying the weight coefficients of the neural units in each hidden layer. Through the continuous adjustment of forward propagation and backward propagation, the output error can be gradually reduced to the desired value(Tang et al., 2013).
2. Naïve Bayes is an algorithm based on Bayesian principles and specialized in dealing with classification problems(Langarizadeh & Moghbeli, 2016). In practice, most combinations of attribute values either do not appear in the training data or do not appear in sufficient numbers. Therefore, directly estimating the multivariate probability of each correlation is unreliable; however, Naïve Bayes assumes that the conditional probability distributions are independent and therefore circumvents this problem(Chen et al., 2020).


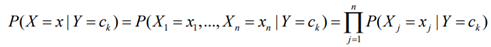
 (1)

Naïve Bayes classifies a given input x and then finds its posterior probability distribution
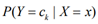
, The maximum a posteriori probability category is used as the output term.A posteriori probability is known according to Bayes' theorem:


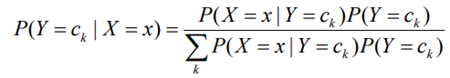
 (2)

The basic formula for Naïve Bayes is obtained by bringing (1) as well as the full probability formula into (2):


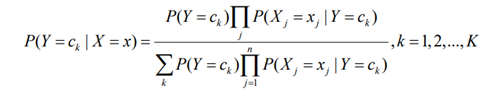
 (3)

Thus Naïve Bayes can be expressed as:


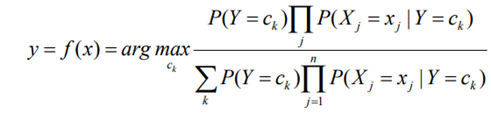
 (4)

And because the denominator is the same for all c_k_, then:


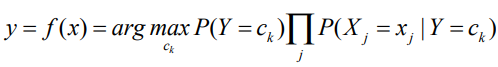
 (5)

1. SVM is a supervised learning algorithm that has become a widely used classification tool, occasionally used to perform regression. The aim is to find a hyperplane that separates two types of data by maximizing the margin. It can handle both simple linear classification tasks and more complex nonlinear classification problems. Many observations often need to be transformed before they can be separated by hyperplanes, and the SVM algorithm transforms the data by using a "kernel function". The kernel function projects the original data points from the input space to a higher dimensional space, or even an infinite dimensional feature space, thus the input variables are separable in the higher dimensional space(Luts et al., 2010). After the kernel function transformation, the optimal hyperplane maximizes the separation between different classes while tolerating misclassification at the formulation level(Noble, 2006).

Cai-Hong, L. J. J. o. X. a. T. U. (2012). BP Neural Network Learning Algorithm. http://dx.doi.org/10.3969/j.issn.1673-9965.2012.09.008

Chen, S., Webb, G. I., Liu, L., & Ma, X. J. K.-B. S. (2020). A novel selective naïve Bayes algorithm. *192*, 105361.

Langarizadeh, M., & Moghbeli, F. J. A. I. M. (2016). Applying naive bayesian networks to disease prediction: a systematic review. *24*(5), 364. http://dx.doi.org/10.5455/aim.2016.24.364-369

Luts, J., Ojeda, F., Van de Plas, R., De Moor, B., Van Huffel, S., & Suykens, J. A. J. A. c. a. (2010). A tutorial on support vector machine-based methods for classification problems in chemometrics. *665*(2), 129-145. http://dx.doi.org/10.1016/j.aca.2010.03.030

Noble, W. S. J. N. b. (2006). What is a support vector machine? *, 24*(12), 1565-1567.

Tang, J., Wu, L., Huang, H., Feng, J., Yuan, Y., Zhou, Y., . . . Yu, C. J. N. R. R. (2013). Back propagation artificial neural network for community Alzheimer's disease screening in China. *8*(3), 270. http://dx.doi.org/10.3969/j.issn.1673-5374.2013.03.010

Wang, B., Tian, P., Sun, Q., Zhang, H., Han, L., & Zhu, B. J. H. (2023). A novel, effective machine learning-based RNA editing profile for predicting the prognosis of lower-grade gliomas. *9*(7). http://dx.doi.org/10.1016/j.heliyon.2023.e18075
