# Supplementary material for: Genomic and algorithm-based predictive risk assessment models for benzene exposure
Source: Front Public Health. 2025 Jan 21;12:1419361. doi: 10.3389/fpubh.2024.1419361 (PMC11795664; doi:10.3389/fpubh.2024.1419361)
Supplement: Supplementary file 3 [file Supplementary_file_3.docx]

**Genomic and Algorithm-Based Predictive Risk Assessment Models for Benzene Exposure**

Minyun Jiang^a ,1^, Na Cai^a,1^, , Juan Hu^e^, Lei Han^b,c,^ , Fanwei Xu^e^, Baoli Zhu^a,b,c,d, ,*^ Boshen Wang^b,*^

1. School of Public Health, Nanjing Medical University, Nanjing 210000, Jiangsu China
2. Institute of Occupational Disease Prevention, Jiangsu Province Center for Disease Prevention and Control, Nanjing 21000, Jiangsu, China.
3. Jiangsu Preventive Medical Association, Nanjing 210000, Jiangsu, China;
4. Center for Global Health, School of Public Health, Nanjing Medical University, Nanjing 210000, Jiangsu, China
5. Southeast University, Nanjing 210009, Jiangsu, China;

* Correspondence to: Baoli Zhu, School of Public Health, Nanjing Medical University, Nanjing 210000, Jiangsu China. Tel: 025-83759982, Fax: 025-83759310, E-mail: [zhublcdc@sina.com;](mailto:zhubl@jscdc.cn;) Boshen Wang, Institute of Occupational Disease Prevention, Jiangsu Provincial Center for Disease Prevention and Control. No. 172 Jiangsu Road, Nanjing, Jiangsu, China. Tel: +86 17766076325, E-mail: [doudouwbs@126.com](mailto:doudouwbs@126.com)

^1^ Minyun Jiang, Na Cai: These authors contributed equally to this work and share first authorship: Minyun Jiang, female, born in 1998, MPH student, majoring in public health; Na Cai, male, born in 1999, MPH, majoring in public health.

Table 1. Basic characteristics of the population

| Variables | benzene-exposed group（n=214) | | benzene non-exposed group (n=231) | | p |
| --- | --- | --- | --- | --- | --- |
|  | n | % | n | % |  |
| Age^a^ | 35（28-47） | | 36（30-46） | | 0.376 |
|  |  | |  | |  |
| sex^a^ |  |  |  |  | 0.399 |
| Male | 156 | 72.90% | 160 | 69.30% |  |
| Female | 58 | 27.10% | 71 | 30.70% |  |
| Smoking^a^ |  |  |  |  | 0.406 |
| Now | 90 | 42.10% | 82 | 35.50% |  |
| Ever | 112 | 52.30% | 145 | 62.80% |  |
| Never | 12 | 5.60% | 4 | 1.70% |  |
| Drinking^a^ |  |  |  |  | 0.603 |
| Now | 81 | 37.90% | 77 | 33.30% |  |
| Ever | 122 | 57% | 149 | 64.50% |  |
| Never | 11 | 5.10% | 5 | 2.20% |  |

1. Non-parametric rank sum test was used
2. Independent samples t-test was used

Table 2. Statistical analysis of the abnormal blood group versus the normal blood group

|  | Blood Abnormalities Group（n=21) | Normal blood group（n=193) | p-value |
| --- | --- | --- | --- |
| NFKB1^a^ | 4.789(2.961-6.038) | 2.215(1.659-3.725) | ＜0.001 |
| PHACTR1^a^ | 2.075(0.848-2.830) | 0.970(0.219-1.899) | 0.004 |
| PTGS2^b^ | 0.997±0.501 | 1.237±0.714 | 0.136 |
| PTX3^a^ | 1.903(1.577-2.140) | 2.272(2.003-2.847) | ＜0.001 |

1. Non-parametric rank sum test was used
2. Independent samples t-test was used

Table 3. Comparative analysis of NFKB1 and PTX3 in smoking status

|  | Now(n=172) | Ever(n=257) | Never(n=16) | p-value |
| --- | --- | --- | --- | --- |
| NFKB1^a^ | 1.56(0.77-2.69) | 1.69(0.52-2.91) | 3.38(1.60-5.57) | 0.073 |
| PTX3^a^ | 2.02(0.84-2.64) | 1.82(0.94-2.25) | 2.20(1.89-2.53) | 0.020 |
| wbc^a^ | 6.69(5.82-7.72) | 6.12(5.16-7.14) | 6.40(5.09-6.91) | ＜0.001 |
| rbc^b^ | 5.07±0.41 | 4.79±0.50 | 4.88±0.24 | ＜0.001 |
| neutrophi^a^ | 3.64(3.01-4.40) | 3.44(2.79-4.28) | 3.46(2.01-3.66) | 0.097 |
| plt^a^ | 213(177-254) | 223(188-261) | 196.5(161.25-217.50) | 0.036 |

a. Non-parametric rank sum test was used

b. Independent samples t-test was used

Table 4. Comparative analysis of NFKB1 and PTX3 in the gender

|  | Male | Female | p-value |
| --- | --- | --- | --- |
| NFKB1^a^ | 1.57(0.57-2.73) | 1.79(0.59-3.32) | 0.159 |
| PTX3^a^ | 1.97(1.03-2.56) | 1.76(0.87-2.16) | 0.01 |
| wbc^a^ | 6.47(5.74-7.46) | 5.95(4.59-7.44) | ＜0.001 |
| rbc^b^ | 5.09±0.38 | 4.45±0.39 | ＜0.001 |
| neutrophi^a^ | 3.53(2.94-4.24) | 3.44(2.64-4.45) | 0.368 |
| plt^a^ | 212.50(177.25-247.75) | 229.00(199.00-276.00) | ＜0.001 |

a. Non-parametric rank sum test was used

b. Independent samples t-test was used

Table 5. The indices of control mice and benzene-exposed mice changed after 4 weeks

|  | Benzene exposure group | Benzene non-exposed group | p-value |
| --- | --- | --- | --- |
| S-PMA^a^ | 53.501（33.290-79.867） | 0.536（0.232-1.199） | ＜0.001 |
| 8-OHdG^a^ | 3.531（2.699-4.391） | 2.347（2.054-2.921） | 0.012 |
| MDA^a^ | 11.206（6.630-18.830） | 5.930（3.551-7.034） | 0.004 |
| WBC^a^ | 0.835（0.628-1.510） | 4.245（3.280-5.088） | ＜0.001 |
| ANC^a^ | 0.445（0.313-0.525） | 0.795（0.543-1.135） | 0.001 |
| PLT^a^ | 438.500（413.250-514.250） | 487.000（460.000-559.500） | 0.089 |
| NFKB1^a^ | 2.444（1.451-3.207） | 1.153（0.473-1.505） | 0.001 |
| PHACTR1^a^ | 1.295（0.923-1.625） | 1.089（0.804-1.330） | 0.347 |
| PTX3^a^ | 2.203（1.341-4.025） | 0.926（0.874-1.420） | 0.001 |
| PTGS2^b^ | 1.62±1.06 | 1.26±0.58 | 0.319 |

1. Non-parametric rank sum test was used
2. Independent samples t-test was used

**Abbreviations**

| 8-OHdG | 8-hydroxy-deoxyguanosine |
| --- | --- |
| AUC | Area Under Curve |
| BP | biological process |
| CC | cell components |
| DEGs | differentially expressed genes |
| GEO | Gene Expression Omnibus |
| GO | Gene Ontology |
| GSEA | Gene Set Enrichment Analysis |
| GSVA | Gene set variation analysis |
| KEGG | Kyoto Encyclopedia of Genes and Genomes |
| LASSO | Least absolute shrinkage and selection operator |
| MDA | Malondialdehyde |
| MF | molecular function |
| PCA | Principal components analysis |
| RF | Random Fores |
| ROC | receiver operating characteristic curve |
| ROS | reactive oxygen species |
| S-PMA | S-phenylmercapturic acid |
| SVM-RFE | Support Vector Machine-Recursive Feature Elimination |
| C5.0DT | C5.0 Decision Tree |
